# Strong Environmental Filtering Based on Hydraulic Traits Occurring in the Lower Water Availability of Temperate Forest Communities

**DOI:** 10.3389/fpls.2021.698878

**Published:** 2022-01-20

**Authors:** Jiale Zhao, Yuhan Zhang, Jinshi Xu, Yongfu Chai, Peiliang Liu, Ying Cao, Cunxia Li, Qiulong Yin, Jiangang Zhu, Ming Yue

**Affiliations:** ^1^Key Laboratory of Resource Biology and Biotechnology in Western China, Northwest University, Xi’an, China; ^2^Xi’an Botanical Garden of Shaanxi Province/Institute of Botany of Shaanxi Province, Xi’an, China; ^3^Guizhou Provincial Key Laboratory for Biodiversity Conservation and Utilization in the Fanjing Mountain Region, Tongren University, Tongren, China; ^4^School of Ecology and Environment, Northwestern Polytechnical University, Xi’an, China; ^5^Shuanglong State-Owned Ecological Experimental Forest Farm of Qiaoshan State-Owned Forestry Administration of Yan’an City, Yan’an, China

**Keywords:** community assembly, environmental filtering, null model, hydraulic traits, Loess Plateau

## Abstract

The trait-based approaches have made progress in understanding the community assembly process. Here, we explore the key traits that may shape community assembly patterns of the same community type but within different water availabilities. Natural *Quercus wutaishanica* forests were chosen as a suitable study system to test the difference between economic and hydraulic traits across water availability on the Loess Plateau (LP, drought region) and Qinling Mountains (QL, humid region) of China. A total of 75 plots were established separately in two sites, and 12 functional traits (seven hydraulic traits and five economic traits) of 167 species were studied. Community-weighted mean trait values and functional diversity indices were compared between the two sites. Canonical component analysis was performed to infer whether the changes of community traits and their relationships are driven by intraspecific variation or species turnover. Evidence for likely community assembly processes was tested using the null model to determine whether functional structure among seven hydraulic traits and five economic traits was dominated by different ecological processes between two sites. We found that forests in the Loess Plateau and Qinling Mountains showed different hydraulic and economic traits. Hydraulic and economic traits coupled at the community level were driven by species turnover. Hydraulic traits showed more significant convergent patterns on LP than that in QL. Our results suggest a strong environmental filtering process occurred in hydraulic-based community assembly in the temperate forest with low water availability. Reveal the relationship of hydraulic and economic traits at the community level. Emphasize the critical role of multi-dimensional traits selecting like hydraulic traits in community ecology.

## Introduction

Community assembly is one of the essential topics in ecology, helping to explain researchers understanding species coexistence and distribution (Ellner et al., 2019). Assembly processes shape the structure and composition of communities (Thorn et al., 2016). Two main theories, neutral theory and niche theory (i.e., limiting similarity and environmental filtering), are commonly invoked to explain the mechanisms of forest community assembly. Based on traits, a community may be characterized by the distribution of species functional traits that compose it (Ackerly and Cornwell, 2007). Because of the direct link between traits and the plant organism’s function, trait strcucture patterns provide significant insights into how communities are assembled (HilleRisLambers et al., 2012; Kunstler et al., 2016; Loranger et al., 2016).

Plant functional traits are the key characteristics to reflect the adaptability of plants to the environment (Targetti et al., 2013). Plants can acquire and invest resources in different ways, the diversity of these strategies has a significant impact on species composition and ecosystem function (Loreau, 2001; Targetti et al., 2013). Therefore, as a link between species and the environment (Lamanna et al., 2014), functional traits are a new perspective to study complex ecological processes. Among various functional traits, those related to water transport and CO_2_ exchange have received more attention, reflecting the crucial importance of processes in the biosphere’s functioning (Li S. et al., 2015). Since the concept of leaf economic spectrum was put forward (LES) (Wright et al., 2004), some traits related to carbon economy have been extensively studied, forming economic traits (Wright et al., 2005). Certain other groups of traits, indicating a balance between the demand and supply of water, form the hydraulic traits (Zhang et al., 2012; Sack and Scoffoni, 2013; Li L. et al., 2015; Yin et al., 2018).

However, at the community level, it is still a new perspective to distinguish hydraulic traits from economic traits, which can provide a basis for revealing the importance of multi-dimensional traits in community assembly study. Liu has considered stomatal traits as hydraulic traits in community assembly study, but more hydraulic traits are not involved (Liu et al., 2020). In addition, current studies have included only economic traits by using the “Null model” (Laughlin and Laughlin, 2013). In the subalpine forest community, limiting similarity is more important for determining species coexistence by selecting specific traits like SLA (Yan et al., 2012). Limiting similarity also shapes the functional structure of plant root traits (Luo et al., 2021). By contrast, soil characteristics could improve environmental filtering effects on some economic traits like leaf dry matter content (Mori et al., 2021). Environmental filtering is the main driving force affecting community functional traits through the study of 15 common economic traits in the Amazon rainforest (Fortunel et al., 2014). Given the large difference of environmental traits among ecosystems, more types of functional traits are needed to better explain the community assembly mechanism.

Understanding how the environment influences the species distribution is a central topic in ecology (Condit, 2000; Baltzer et al., 2009). Environmental factors, such as water availability, play a critical role in species distribution patterns and can work as filters that shape plant communities’ function or composition (Groom, 2004; Engelbrecht et al., 2007; Moeslund et al., 2013). In order to adapt to environmental changes, species have developed corresponding adaptive strategies to the local environment (Liu et al., 2021), and these strategies are reflected in functional traits. A previous study suggests that the water availability changes in Loess Plateau forests involve trade-offs between economic and hydraulic traits, and that this correlation of leaf economics and hydraulic traits might be a type of adaptation mechanism (Yin et al., 2018). In habitats with low water availability, plants must invest resources in organs that can improve hydraulic resistance (Rowland et al., 2015), which leads to changes in hydraulic traits. For example, the increase of sapwood area, can reduce water loss to the greatest extent (Gotsch et al., 2019), minimize photosynthesis, and growth rate (Delzon et al., 2004). Trade-offs among different strategies produced by plants in response to stress are one important mechanism of coexistence (Silvertown et al., 2015; Letten et al., 2017). Hydraulic traits also constrain plant distributional ranges (Li et al., 2018). The previous studies mentioned above revealed that plants produce different trait combinations to adapt to the local environment. At the species level, the relationship between economic and hydraulic traits is well explored (Sack and Scoffoni, 2013). Furthermore, Liu discussed the variation in leaf morphological, stomatal, and anatomical traits (Liu et al., 2019). Li discussed the leaf economic and hydraulic traits relationships in five tropical-subtropical forests (Li S. et al., 2015). Therefore, in habitats with different water availability, it can be speculated that the relative importance of the two kinds of traits at the community level is different. The combination between hydraulic and economic traits can be used as a filter factor to affect the coexistence of species and community assembly patterns, but this still requires direct evidence to verify.

Since the Middle Pleistocene, *Q. wutaishanica* has been one of the dominant tree species in deciduous broad-leaved forests of northern China. The forest communities dominated by *Q. wutaishanica* are climax vegetation occupying the upper limit of deciduous broad−leaved forests in the intermediate altitude of Qinling Mountains (QL) and the northwestern limit (related to water availability) of forests on the Loess Plateau (LP) (Zhu, 1993). The *Q. wutaishanica* community on the Loess plateau differed from many other forests of their kind due to the wide range of water limitations they are facing. This special *Q. wutaishanica* forest distribution pattern and environment type provide an excellent opportunity to explore the changes of plant functional traits and assemblages of communities with the same evolutionary history under different water use conditions.

In this study, we aim to discuss the differences and effects of economic and hydraulic traits at the community level and explore the pattern of community assembly within different water availability of *Q. wutaishanica* forests by using economic and hydraulic traits separately. We hypothesized that (1) forests growing in water-rich environments will have traits related to resource acquisition, but forests growing in water-poor environments will have traits related to resource conservation. (2) Economic and hydraulic traits are strongly coordinated at the community level. (3) Functional diversity and economic traits reflect the different ecosystem function within different water availabilities. (4) The community assembly process is different based on hydraulic traits from economic traits. Carbon and light competition would limit economic trait similarity to avoid competitive exclusion and facilitate niche partitioning in two sites. While drought environment characterizing lower water availability may lead to stronger environmental filtering in hydraulic traits.

## Materials and Methods

### Study Site and Sampling

This study was conducted in a well-protected typical *Q. wutaishanica* forest in Shaanxi province, China. The study was performed in two sites at the Ziwuling region (35°41′-35°44′ N, 109°00′-109°02′ E) on the middle of Loess Plateau and the Taibaishan Nature Reserve (33°84′-33°86′ N, 108°82′-108°87′ E) in the north slope of Qinling Mountains. The *Q. wutaishanica* forest in the Qinling Mountains has a temperate monsoon climate, which is relatively humid (annual precipitation 900–1,000 mm), but the Loess Plateau has a temperate continental climate, which is relatively dry and going through serious water erosion (annual precipitation 550–650 mm), such climatic conditions lead to direct differences in soil water content (Supplementary Figure 1).

The field work was surveyed in July 2019. Three permanent plots covering an area of 2,500 m^2^ (50 m × 50 m) were established in the two study areas, respectively, to investigate this research (Table 1). The distance between any two plots was more than 1 km to avoid spatial auto correlation in variables and pseudo-replications (Conti and Dıaz, 2013). Each plot was subdivided into 75 quadrats of 10 m × 10 m. All woody species in plots were identified within each plot and their richness (the number of plants) was measured as the basic community data.

**TABLE 1:** Plot setting and geographic information.

	Administrative region	Coordinate of latitude and longitude	Area (m^2^)	Elevation (m)
Qinling Mountains	Meixian county	107°45′E/34°41′N	2,500	1,958
	Meixian county	107°45′E/34°20′N	2,500	1,937
	Jiwozi village	108.83°E/33°84′N	2,500	1,900
Loess Plateau	Huangling county	109°01′E/35°42′N	2,500	1,100
	Huangling county	109°00′E/35°49′N	2,500	1,112
	Huangling county	109°11′E/35°37′N	2,500	1,100

Soil water content (SWC) was using the oven drying method. Three sites were randomly selected in each plot. The external humus was stripped away, exposing the topsoil. Soil samples of 0–10 cm and 10–20 cm were drilled with a metal sampler. A total of six soil samples was obtained in each plot. After drilling the soil, gravel and plant roots were removed, and the portable electronic balance was used to weigh the fresh mass of the soil, which was then brought back to the laboratory to be dried at 105°C oven to constant weight as the dry mass, so as to calculate the soil water content.

Leaves were collected from woody species in every plot. For each species, at least three individuals were sampled and stored separately. Mature and fully expanded leaves were collected from the sun-exposed branches of each individual. After harvesting, 30 leaves of each species were preserved in black plastic bags to protect from light for analyses of economics traits, 3–5 leaves and branches were maintained in a formalin acetic alcohol (FAA; 5 ml of 38% formalin, 5 ml of glacial acetic acid, 90 ml of 50% ethanol, and 5 ml of glycerin) for analyses of hydraulic traits like venation traits, stomatal traits, and anatomical structures (Chen and Wang, 2009).

### Trait Selection

Here, we measured 12 functional traits of 167 woody plants (Supplementary Figures 2, 3) which grow in Q. wutaishanica forests on LP and in QL. Four of these traits were considered as economic traits, which are correlated with nutrient acquisition, light acquisition, and biomass allocation in plants, eight of these traits were considered as hydraulic traits, which are correlated with water transport and transpiration in plants (Table 2).

**TABLE 2:** Functional traits employed in this study as well as their abbreviations, units, and functions.

	Plant traits	Abbreviations	Units	Function
Economic traits	Leaf dry mass per area	LMA	g/m^–2^	Resource capture
	Leaf dry matter content	LDMC	mg/g	Leaf structure
	Leaf thickness	LT	μm	Resistance to disturbance
	Leaf tissue density	TD	G cm^–3^	Leaf defensive
	Maximum net photosynthetic rate	P_max_	mmol m^–2^. s^–1^	Leaf cooling
Hydraulic traits	Huber value	Hv		Water transport
	Maximum vessel diameter	Vd_max_	μm	Resistance to embolism
	Stomatal density	SD	mm^–2^	CO_2_ acquisition and water loss
	Stomatal length	SL	μm	CO_2_ acquisition and water loss
	The leaf turgor loss point	Ψ_tlp_	Mpa	Tolerance to drought stress
	Branch wood density	WSG_branch_	g/cm^–3^	Water transport
	Vein density	VD	mm mm^–2^	Water distribution strategy

#### Economic Traits

First, a photograph of each fresh leaf surface was taken with a digital camera, and leaf surface area was measured with Motic Images Plus 6.0 (Motic China Group, Xiamen, China) software. The fresh leaf mass was measured with electronic balance (one ten-thousandth). Then all leaves were placed in a drying oven for 72 h at 70°C to determine the dry mass. Leaf dry mass per area (LMA, g m^–2^) was calculated as the ratio of the leaf dry mass to leaf surface area. Leaf dry matter content (LDMC, mg/g) was calculated as the ratio of the leaf dry mass to leaf fresh mass. Leaf thickness (LT, mm) was measured through transverse sections using Image-Pro Plus 6.0, avoiding the influence of major veins. For each section on one leaf, 10–20 measurements were made. Leaf tissue density (TD, g cm^–3^) was calculated as the ratio of LMA to LT. Net photosynthesis rates of mature and fully expanded leaves were measured between 9:00 and 11:00 with the portable photosynthesis system (Li-6800, Li-Cor, Lincoln, NE). CO_2_ concentration was that of the ambient air and the flow rate was set at 500 μmol s^–1^. Light was provided with an LED and the photosynthetically active radiation (PAR) gradients were 1,800, 1,500, 1,200, 1,000, 800, 600, 400, 200, 100, 50, 20, and 0 μmol m^–2^ s^–1^, and the maximum net photosynthetic rate was obtained by fitting the empirical equation of the least square method (Bassman and Zwier, 1991).

#### Hydraulic Traits

We used the nail-polish imprint method to measure stomatal traits on three leaves for one individual and three individuals for one species (Zhao et al., 2016). We photographed the stomatal prints under a Classica SK200 digital light microscope (Motic ChinaGroup Co., Ltd., China). Then we used Image-Pro Plus 6.0 software to measure SL (μm). SD (mm^–2^) was calculated as the number of stomata per unit leaf epidermal area by dividing leaves into grids of 100 μm × 100 μm. SL and SD were averaged from more than 20 randomly selected fields of view. Water potential was measured in consecutive sunny days of mid-July 2019 with a pressure chamber (SEC3115, Santa Barbara, CA, United States). Leaf samples from three individuals of each species were cut from trees and sealed immediately in black plastic bags with a moist paper towel in them and kept in a cooler during the transportation (about 30 min) to the laboratory. Leaves were first weighed to obtain the initial fresh mass and then immediately placed in a pressure chamber to determine the initial water potential. Leaf weight and water potential were measured periodically during slow desiccation of the sample in the natural condition. Finally, leaves were oven-dried for 72 h at 70°C to determine the dry weight. The leaf turgor loss point (Ψ_tlp_, MPa) was calculated by the PV curve method based on the natural wind dry method (Tyree and Hammel, 1972).

We selected three complete annual branches for one individual with good light conditions on the upper part of the species. Branches that were 5 cm long were cut from the base of the branches to remove the bark and determine the wood density (WSG_branch_). The volume of the branches was obtained by the drainage method, followed by oven-drying at 70°C for 72 h before weighing. The dry weight of the branches was obtained by weighing scales with an accuracy of 0.0001 g. The branch wood density is the dry weight of the annual branch divided by the volume of the fresh branch.

The diameter of the branches was measured by a vernier caliper (μm). Heartwood and pulp were subtracted, and the sapwood area [As_(branch)_] of the branches was obtained. At the same time, all the leaves on the branches were collected, and then the ratio of sapwood area to total leaf area was calculated as the Huber value (Hv).

We took leaves from the FAA and cut complete leaf petioles, and the transverse sections were made on a Leica RM2135 rotary microtome (Leica Inc., Bensheim, Germany) and then mounted on glass slides. The transverse sections were observed and photographed under an optical microscope, and the images were measured using Image-Pro Plus 6.0 (Media Cybernetics, Unite States) software to get the maximum vessel diameter (Vd_max_, μm^2^).

For vein density (VD, mm mm^–2^) assessments, leaves were sampled about 2 cm^2^ in the central region, immersed in 10% NaOH in an oven at 65°C for 4–12 h. Samples were repeatedly washed with deionized water for about 30 min, immersed and bleached in 10% H_2_O_2_ for 10–30 min, and then washed again in deionized water. Samples were next stained with safranin for 30 min. Thereafter, they were dehydrated using graded ethanol series (30, 50, 70, 85, 95, and 100%) and immersed in xylene: ethanol absolute (1:1) solution and xylene. Stained sections were mounted, photographed, and then analyzed using Image-Pro Plus 6.0. The total length of veins per unit area was measured as VD.

### Statistical Analyses

#### Community Weighted Mean Value

We examined correlations between weighted traits of communities. The community-weighted mean (CWM) of all 12 traits was calculated for each plot as a weighted average of species traits, average trait value from plots with weightings based on species relative abundance (trees ≥ 10 cm Diameter at Breast Height) (Hulshof et al., 2013):


$$\text{CWM}=\sum_{i}^{n}p_{i}Trait_{i}$$

where n was the number of species sampled in the plant community, p_i_ was the relative abundance of i^th^ species, and Trait_i_ was the mean trait value of the i^th^ species. Significant differences in CWM values from two sites for the whole 12 traits were tested by the Student’s t-test. In order to obtain comprehensive trait values in multiple dimensions, multivariate associations of traits were analyzed with a canonical correlation analysis (CCA) in CANOCO software for Windows 5.0 (Microcomputer Power, Ithaca, NY, United States) and inductive analysis the traits represented by the first two axis of CCA. Plant community data consisted of the relative abundance of 167 species in two sites, respectively.

#### Community Functional Diversity

We assessed the effects of species traits on ecosystem functioning by computing three kinds of functional diversity indices. Functional richness (FRic) is the volume delimited by the smallest convex hull drawn around the existing species positioned on trait axes according to their trait values and reflects the amount of niche space occupied by the community. One-dimensional functional richness (FDis) is the ratio of the trait range of a species to the trait range of all species in the community (Schleuter et al., 2010). Functional divergence (FDiv) is the variance of species trait distribution in the trait space and functional evenness (FEve) is the degree to which the biomass of a community is distributed in niche space (Mason et al., 2005). Rao’s quadratic entropy (RaoQ) is defined to measure the diversity and difference within and between populations (Botta-Dukát, 2005). All indices were calculated within the “FD” package (Laliberté) in R (Version 4.02; R Core Team, 2015).

#### Community Functional Structure by Null Model

Ecological null model approaches are the most frequently used tools for studying the assembly process because these are appropriate for the identification of non-random components in community composition. To assess if functional traits obtained per plot differed from random, we used a null model approach to deduce assembly processes (Ricotta and Moretti, 2011).

Different vertical structures have different strategies in Q. wutaishanica communities, which may lead to different community assembly processes (Chai et al., 2016). Functional structure of the tree layer and the whole woody community were calculated, respectively, to explore the differences at different vertical structures based on economic and hydraulic traits.

The essence of the approach is that trait divergence or convergence is characterized by a test statistic and this test statistic was run for 999 random samples created from the species pool. Species pools were established in QL and on LP, respectively. Specifically, a wide range of species were sampled according to the Flora of Loess Plateau and Qinling Mountains to expanded species pools. The test statistic was also calculated for the real samples, and proportions of random communities where the test statistic is more extreme than in the field sample. This study measured the “mean functional distance” (MFD) of species in the community to calculate the value of “standard effect size values” (SES). We used probit transformed p-values as SES, which indicate the strength of trait divergence or convergence. Positive SES values indicate that competition is the leading assembly rule, while negative ones refer to the leading role of environmental filtering. Significant differences in SES values from zero for the whole dataset were tested by the Student’s t-test. The standard effect size of all traits was calculated by the “Picante” package (Kembel) of R software.


$$\mathrm{Standardized}\mathrm{effect}\mathrm{size}\left(\mathrm{SES}\right)=\frac{{\text{MFD}}_{\text{observed}}-{\text{MFD}}_{\text{randomized}}}{{\text{sdMFD}}_{\text{randomized}}}$$

where MFD_observed_ is the observed value, MFD_randomized_ is the mean of the simulated values, and sdMFD_randomized_ is the standard deviation of the simulated values.

## Results

### Community Weight Mean Value Between Two Sites

The CWM varied across plots between LP and QL. Eleven of twelve CWM traits differed significantly between the LP and QL (the abbreviations and function of traits are shown in Table 2).

**FIGURE 1:**
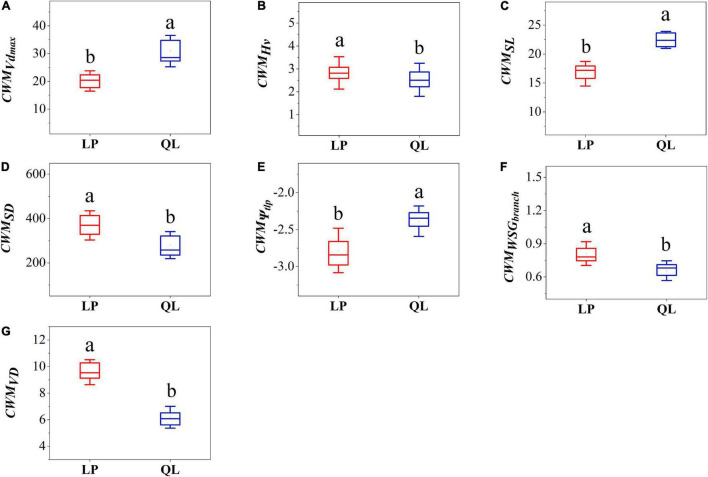
Box-plot comparing community-weighted mean value of **(A)** maximum vessel diameter, **(B)** Huber value, **(C)** stomatal length, **(D)** stomatal density, **(E)** the leaf turgor loss point, **(F)** branch wood density, **(G)** vein density between LP and QL. Different letters indicate significant differences (*P* < 0.05).

**FIGURE 2:**
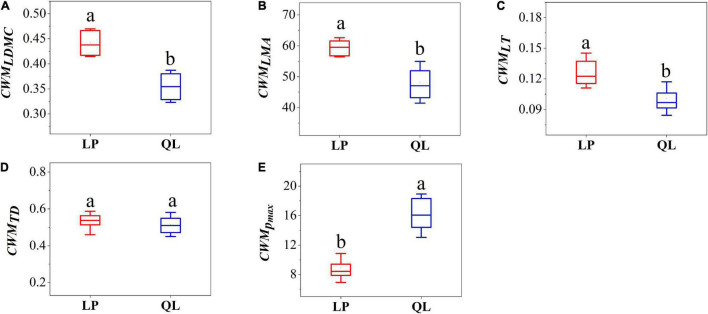
Box-plot comparing community-weighted mean value of **(A)** leaf dry matter content, **(B)** leaf dry mass per area, **(C)** leaf thickness, **(D)** leaf tissue density, **(E)** maximum net photosynthetic rate between LP and QL. Different letters indicate significant differences (*P* < 0.05).

In the analysis of CWM hydraulic traits, communities on the Loess Plateau had significantly higher Hv, SD, WSG_branch_, and VD compared to those from QL (Figures 1B,C,F,G). Communities from QL had significantly higher SL and Vd_max_ than those of the Loess Plateau (Figures 1A,D,E); In the analysis of CWM economic traits, communities on the Loess Plateau had significantly higher LDMC, LT, and LMA compared to those from Qinling Mountains (Figures 2A,B,C). Communities from the QL had significantly higher P_max_ than those of the LP (Figure 2E). TD showed no significant differences between the LP and QL (Figure 2D).

**FIGURE 3:**
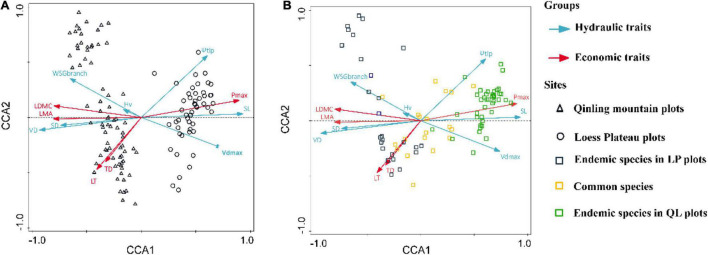
Community-weighted trait bi-plot for canonical correlation analysis, showing the effects of functional traits on the community species composition and plot distribution between the Loess Plateau and Qinling Mountains. **(A)** The effect of functional traits on the plot distribution. **(B)** The effect of functional traits on the community species composition. The ordination is based on community-weighted mean traits, with traits weighted for each species by its relative abundance in the community. Trait abbreviations are given in Table 1.

The first axis of the CCA of 12 traits explained 33.4% of the relation. Three hydraulic traits (SD, VD, SL) and three economic traits (LDMC, LMA, P_max_) were strongly associated with the CCA1 axis (Table 3). We also found that plots and species distributions had a significant difference between LP and QL (Figure 3). Different species were grouped based on water availability along the CCA1, with QL and LP plots well separated. Generally, the plots in LP were grouped, whereas those plots in QL were grouped together (Figure 3A). Generally, the CCA tests showed that the hydraulic and economic traits were coupled on the community level, and the species composition showed a certain regularity along with water availability at a regional scale.

**TABLE 3:** Loadings of measured traits to CCA1 and CCA2.

Traits	CCA1	CCA2
Stomatal length (SL)	0.9091	0.0264
Vein density (VD)	–0.9088	–0.0969
Maximum net photosynthetic rate (P_max_)	0.8763	0.1314
Leaf dry mass per area (LMA)	–0.7877	–0.0306
Leaf dry matter content (LDMC)	–0.7805	0.0872
Stomatal density (SD)	–0.7217	–0.0805
Maximum vessel diameter (Vd_max_)	0.7192	–0.2473
Branch wood density (WSG_branch_)	–0.6381	0.3060
The leaf turgor loss point (Ψ_tlp_)	0.5925	0.4848
Leaf thickness (LT)	–0.3954	–0.4121
Leaf tissue density (TD)	–0.3192	–0.3447
Huber value (Hv)	–0.1593	0.0806

### Community Functional Diversity Indices

**FIGURE 4:**
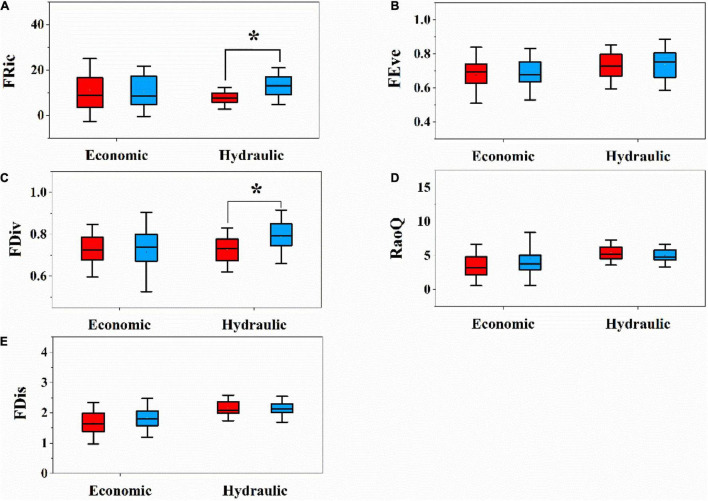
Difference of functional diversity components in relation to hydraulic traits and economic traits between LP and QL. **(A)** Functional richness (FRic); **(B)** functional evenness (FEve); **(C)** functional divergence (FDiv); **(D)** Rao’s quadratic entropy (RaoQ); **(E)** one-dimensional functional richness (FDis); different letters above bars indicate statistically significant differences at *P* < 0.05.

We analyzed functional diversity indices (FRic, FEve, FDiv, FDis, and RaoQ) in each plot between the LP and QL separately. Only FDic and FDiv based on multiple hydraulic traits significantly showed different patterns between two sites (Figures 4A,C). That is, the QL had higher FDic and FDiv on multiple hydraulic traits. There was no significant difference in other functional diversity indices (FRis, FEve, and RaoQ) based on both multiple economic traits and multiple hydraulic traits between the two sites (Figures 4B,D,E).

### Functional Structures Between Two Sites

**FIGURE 5:**
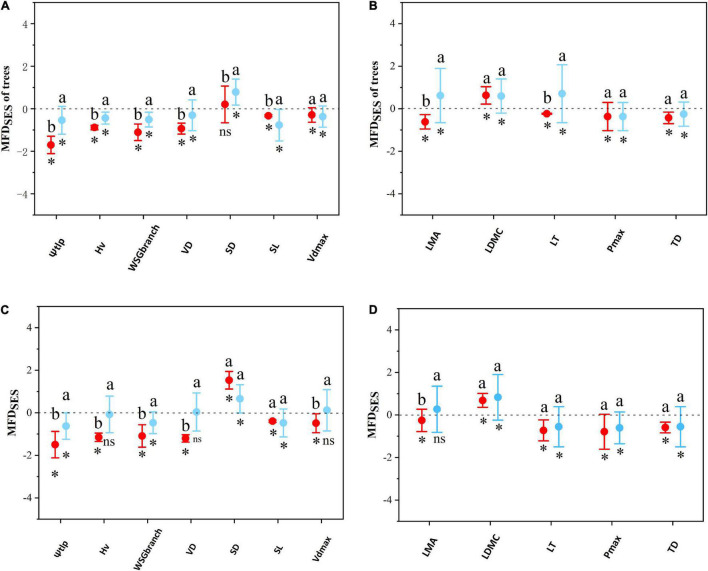
The difference of community functional structures based on single traits between the Loess Plateau (LP) and the Qinling Mountain (QL). The functional structures were measured as standardized effect sizes (SES). **(A)** Test on single hydraulic traits in tree stratum. **(B)** Test on single economic traits in tree stratum. **(C)** Test on single hydraulic traits in the whole site. **(D)** Test on single economic traits in the entire site. Specifically, SES > 0 represents functional divergence, whereas SES < 0 indicates functional convergence. Each point shows the mean SES values among all plots within the LP and the QL. Error bars represent standard errors. Asterisks indicate significant differences between SES with 0. Different letters above bars (ab) indicate statistically significant differences at *P* ≤ 0.05 between the LP and the QL in the same trait.

**FIGURE 6:**
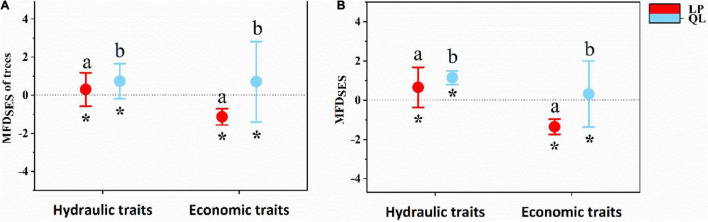
The difference of community functional structure [standardized effect size (SES)] based on multiple traits between the Loess Plateau (LP) and the Qinling Mountain (QL). **(A)** Test on multiple hydraulic and economic traits in tree stratum. **(B)** Test on multiple hydraulic and economic traits in the whole site. Standardized effect size > 0 represents functional divergence, whereas SES < 0 indicates functional convergence. Red point and blue point show the mean SES values within the LP and the QL. Error bars represent standard errors. Asterisks indicate significant differences between SES with 0. Different letters above bars (ab) indicate statistically significant differences at *P* < 0.05 between the LP and the QL in the same trait.

In the tree layer, single-trait analyses indicated SES of most hydraulic and economic traits showed a deterministic pattern between the two sites (Figure 5), except for SD on LP. As for hydraulic traits, functional structures of plant Ψ_tlp_, Hv, WSG_branch_, and SL were more convergent on LP than in QL (Figure 5A). There was no significant difference in functional structures of Vd_max_. As for economic traits (Figure 5B), functional structures of plant LMA and LT were more convergent in QL than on LP. There was no significant difference in the functional structures of LDMC, P_max_, and TD. Multi-trait analyses also indicated that the SES of multiple hydraulic and economic traits showed a deterministic pattern between the two sites (Figure 6A) in the tree layer. Functional structures of both hydraulic and economic traits were more convergent on LP than in QL.

In the whole woody community, similarly, single-trait analyses indicated that the SES of most hydraulic and economic traits showed a deterministic pattern between the two sites (Figure 5), except for Hv, VD, and Vd_max_ in QL. As for hydraulic traits, functional structures of plant Ψ_tlp_, Hv, WSG_branch_, VD, and Vd_max_ were more convergent at LP than in QL (Figure 5C). There was no significant difference between the functional structures of SD and SL; as for economic traits, the functional structure of plant LMA was more convergent in QL than on LP (Figure 5D). There was no significant difference in the functional structures of LDMC, LT, P_max_, and TD. Multi-trait analyses also showed that the SES of multiple hydraulic and economic traits showed a deterministic pattern between two sites (Figure 6B). Functional structures of both hydraulic and economic traits were more convergent on LP than in QL. The results in the entire community were the same as in the tree stratum.

## Discussion

Our investigation demonstrated that a combination of plant hydraulic traits (SD, VD, SL) and economic traits (LDMC, LMA, P_max_) comprises a group of key traits which are important for understanding the community assembly process in *Q. wutaishanica* forests of different water availability. The significant difference in hydraulic community-weighted mean traits indicated that species in LP experience stressful water conditions in comparison with QL. A strong convergent pattern in single and multiple hydraulic traits of LP’s *Q. wutaishanica* forest communities suggests that environmental filtering plays a leading role in the assembly process compared with QL.

### Different Water Use Strategies Between the Two Sites

The shifts in CWM traits (Figures 1, 2) represent plant traits’ responses to stress in the community. In the two *Q. wutaishanica* forests with different water availability, the species within plots showed different functional strategies. We showed that the water availability was a strong power that selected different ecological strategies. LP was dominated by species with conservative hydraulic traits (avoid embolism). While hydraulic traits in QL were mostly associated with acquisitive strategies (improve water supplement efficiency).

For economic traits, we found that with the decrease of water availability from QL to LP, LDMC, LT, P_max_, and LMA increased significantly, while TD showed no significant difference. In the LP, under the limited water resources or relatively dry conditions, other stressful conditions may also gradually increase from QL to LP as soils become barren and canopy cover decreases. In this case, LDMC and LMA are also closely related to water (Cornelissen et al., 2003), and the plants have relatively small and thicker leaves, higher leaf dry matter content, and higher water retention ability than those species distributed under abundant soil resources (Dwyer et al., 2014). These changes are reflecting a strategy to minimize water loss and respiration costs when water is not sufficient which is consistent with previous results (Subedi et al., 2019). On the contrary to LP, the *Q. wutaishanica* forest in QL has sufficient soil moisture (Liu et al., 2017) and is typically accompanied by quick growth with relatively large and soft leaves, relatively high leaf area, and relatively low leaf dry matter content. The weighted average of the net photosynthetic rate at the community level increased with the increase of water availability because photosynthesis also influences plant growth (Garnier et al., 2004), which appears to be a strategy to survive in rich soils (Givnish, 2002) without water limitation “trouble.”

There were also differences in the community weighted mean values of all seven hydraulic traits between the two sites. Under the drought environment, plants must take a conservative strategy by investing resources in traits that can improve hydraulic resistance to ensure water transport safety (Rowland et al., 2015). To minimize the risk of embolism, plants reduce the diameter and increase the number of vascular bundles in the sapwood area (Fortunel et al., 2014). The increase of sapwood area and the decrease of Huber value can reduce water loss to the greatest extent (Gotsch et al., 2019), but at the same time reduce photosynthesis and growth rate (Delzon et al., 2004). Plants in the drought environment also need higher VD to ensure water supply (Sack and Scoffoni, 2013). Besides, small and dense stomata can more flexibly sense changes to drought and close stomata to reduce water loss in a timely manner (Franks et al., 2009). Plants also have low Ψ_tlp_ to maintain certain stomatal conductance, hydraulic conductivity, and photosynthetic gas exchange. These changes reflected a conservative strategy in the drought environment (Lenz et al., 2006; Reich et al., 2007; Bartlett et al., 2012). Instead, the humid environment has filtered out species that invest in improving hydraulic efficiency, which we can define as risk-taking strategies (Hacke et al., 2001; Fan et al., 2011). In our results, species of *Q. wutaishanica* in LP have higher WSG_branch_, Hv, and VD (Figure 1), which allows them to resist drought stress by maintaining high water potentials (Westoby et al., 2002).

The differences in hydraulic and economic traits between the two sites demonstrate the differentiation of ecological strategy in the *Q. wutaishanica* community, and this, in turn, enables them to develop strategies for facing different environments. Within each forest, there is a continuous change in water availability, where low water availability will lead to communities with drought resistance and conservative traits. At high levels of water availability, such a tolerance strategy will not be feasible anymore, leading to communities with resource acquisition and water use efficiency traits. The *Q. wutaishanica* community in QL was more inclined to the efficiency strategy, whereas the *Q. wutaishanica* community on LP was more inclined to the safety strategy. These results in CWM reveal the difference in hydraulic and economic traits between the two sites and provide a background for further discussion of species coexistence and community assembly under different water conditions. More evidence is needed to show the degree to which economic traits and hydraulic traits represent environment filtering.

### The Coupled Relationship Between Economics and Hydraulic Traits Among Community Level

The results of CCA analysis intuitively reflect the differences in CWM of the surveyed community. The study plots were separated along CCA 1, and the species distribution was relatively dispersed, so it could be seen that the differences in traits were caused by species turnover rather than intraspecies variation. At the same time, most of the common species in the two sites were dominant trees (like *Quercus wutaishanica, Betula albosinensis, Toxicodendron vernicifluum*, etc.), and their relationship with CWM is more concentrated. Yin et al. (2018) found coupled relationships between economic and hydraulic traits by using 47 woody plants on the Loess Plateau. Therefore, our results indicate a strong correlation among community-level traits, which resulted in plant communities adopting a cost-effective strategy to improve resource use efficiency and/or resistance to stress in low water availability (Flores-Moreno et al., 2019; Liu et al., 2020). Besides, water availability promoted the turnover of understory shrub species. The coupled relationship between the economic traits and the hydraulic traits among the community level at the regional scale improved the overall adaptability of the *Q. wutaishanica* community.

The CCAs also help us to determine important traits that correlate with aspects of species distribution. The interactions in functional traits, species composition, and plot distribution highlight the importance of trait selection that represents specific strategies for exploring community assembly mechanisms. Therefore, a combination of plant hydraulics and economic traits form a group of key traits which are significant for understanding the community assembly process in *Q. wutaishanica* forests between the two sites. Different assembly processes may be operating simultaneously along with these distinct functional strategies (Spasojevic and Suding, 2012). Therefore, choosing different functional traits (hydraulic and economic) helps us uncover the cause of species turnover and community assembly processes in this area. Ultimately, based on the trait patterns we have found, we suspect that convergent species distribution at LP (lower water availability) may be the result of filtering process for the same traits.

### Functional Diversity Based on Hydraulic and Economic Traits in Response to Water Availability Change

Functional diversity is considered as an important driver of community assembly across environmental gradients (Sanaphre-Villanueva et al., 2016). Thus, the difference in the functional diversity of hydraulic traits between the two sites also reflects the difference in the community assembly pattern. The hydraulic trait functional richness (FRic) of the *Q. wutaishanica* community in the LP was lower, indicating that the community was more affected by environment filtering, which made the species in the community have similar hydraulic traits and reflect the elimination of poor competitors in water acquisition and in line with the negative values of FDiv found there. Relatively high FRic values of hydraulic traits in QL indicates that the community has high ecological space utilization, high niche differentiation of water use among species, and limiting similarity process acts on the coexistence of species (Mason et al., 2012), and the coexistence of acquisitive and conservative species that show different constellations of hydraulic traits (Mayfield and Levine, 2010).

There are also significant differences in hydraulic traits of functional divergence (FDiv) between the two sites. The functional divergence reflects the dispersion degree of functional traits in the community. LP showed negative FDiv, suggesting that environmental filtering may reduce the functional strategies present in this environment, which is also in line with the negative values of FRic found there. Communities with higher functional divergence have more complete ecosystem functions like Qinling Mountains (Xu et al., 2014). The high degree of divergence indicates that plants have a high degree of niche differentiation in environment water utilization, and it may be produced by the direct lower competition. The comparisons of functional diversity in our study verified the difference in the assembly process between hydraulic and economic traits. We found evidence of environmental filtering in LP through hydraulic traits functional indices, probably due to the effect of lower soil water availability.

### The Difference of Community Assembly Process Based on Hydraulic and Economic Traits Between Two Sites

As for multiple hydraulic and economic traits, the null model revealed that community functional structure of QL and LP showed the deterministic processes which dominated the community assembly of both sites (Figures 5, 6). Previously found in the *Q. wutaishanica* community (Wang et al., 2019), trait divergence has been commonly observed (Norden et al., 2012; Purschke et al., 2013). Moreover, this pattern is generally attributed to the elimination of functionally similar species caused by competition or colonization of distantly related species (Li S. et al., 2015). As for single traits, the functional structures for most hydraulic and economic traits showed a deterministic process (Figure 5). Most of the functional traits showed a convergent pattern, indicating that environmental filtering drove the community assembly process in some traits. Several functional traits showed the divergent pattern as well, which indicated that limiting similarity also contributed to the community assembly process of the *Q. wutaishanica* forests in the two sites. Biotic interactions such as competition may work in the divergence of two hydraulic and economic traits (SD, LDMC). One possible reason is that plant stomata are easily modified by environmental conditions and are sensitive to changes in the external environment, resulting in differentiation in SD adapted to concentrations of carbon dioxide in global warming (Wu et al., 2018). This result appears to suggest the limiting similarity process lessens competition among plants, in which resource competition acts to constrain local neighborhoods to certain two traits or trait combinations (SD and LDMC). Based on the different assembly pattern between single and multiple hydraulic traits, we suspected that the competitive interaction among species in SD led to the divergence of overall hydraulic traits functional structure in the two sites. In general, limiting similarity and environmental filtering of single traits may work to counteract each other, resulting in divergence but increasing convergent patterns of multi-hydraulic traits.

The selected economic and hydraulic traits describe the adaptability of species to water resources acquisition, carbon investment, and mechanical support. These different types of functional traits and trait combinations will reflect the adaptation strategies of plants to different environmental conditions (Drenovsky et al., 2012). Consistent with our result, a previous study found that reproductive traits and canopy height showed divergent and convergent patterns, respectively, in the temperate primary forests of northwest Germany (Naaf and Wulf, 2012). Here, traits convergence (environmental filtering) and traits divergence (limiting similarity) can also simultaneously act on different functional traits in response to different biological and abiotic mechanisms, and thus collectively drive community assemblage.

There were shifts in economic and hydraulic functional structure, transitioning from QL to LP with a decrease of water availability. From QL to LP, all 12 measured traits exhibited significant phylogenetic signals (*P* < 0.05, Supplementary Table 1), functional structures of plant hydraulic traits (Ψ_tlp_, Hv, WSG_branch_, Vd_max_, and VD) and an economic trait (LMA) convergent more in LP than in QL. Especially, functional structures based on hydraulic traits from QL to LP clearly showed more convergence except for SD. However, among economic traits, only LMA showed this convergent trend (Figure 2).

*Quercus* spp is the common and dominant genus in climax communities and is particularly abundant in northern China, which represents a strong impact of species abundance on community assembly (Chai et al., 2019). On the LP, natural *Q. wutaishanica* forests experienced serious water erosion (Shangguan et al., 2002), and therefore hydraulic traits are sensitive to water conditions (Xu et al., 2016). Hydraulic traits based functional structure may thus be considered as a consequence of increasing environmental filtering to adapt to drought environment. The coexistence of species in QL did not produce such strong adaption. Although some traits in both sites are driven by the same processes, we determined a novel trend that the functional structure of hydraulic traits is changing toward more convergent from QL to LP. Overall, insignificance with respect to a single economic trait functional structure, a significant hydraulic trait convergent pattern suggested that environmental filtering acting on hydraulic traits was mostly stronger than that acting on economic traits on LP. Such a result supports the expectation that the low water availability characterizing drought environments influences trait values, which filtered out of the regional species pool. Low water availability either led to stronger environmental filtering in hydraulic traits and improved the degree of traits convergence on LP compared with QL. However, unlike what we hypothesized, we found no negative relationship in the mechanism of community assembly between economic traits and hydraulic traits in *Q. wutaishanica* forests.

From a parallel and finer perspective, our CCA results showed that the patterns of species and plot distributions in QL and on LP are different. The observed species grouped on LP and in QL separately. At the same time, considering the same pattern we have found in the tree layer driven by environmental filtering, our results also reflect that the variation in the traits of tree species and the turnover of understory shrub species were the determinants of the different community patterns in the two sites. These two patterns among the species suggested that species on LP were mostly specialists in the local environment, and species’ tolerances for limiting water may be more important than competition for resources. In the study areas, the selected hydraulic traits showed the same assembly process as the economic traits, but the response degree between economic and hydraulic traits was different when considering the environmental condition. Our results showed that the response of hydraulic traits was more sensitive in the community assembly within different water availability, and confirmed the key traits of the local *Q. wutaishanica* forest community assembly process. Moreover, this study also revealed the relative importance of different kinds of traits. Thus, to better understand the mechanisms of communities acclimating to the environment, more representative traits should be considered according to environmental differences (Adler et al., 2013), such as hydraulic traits.

## Conclusion

In summary, by using data of two *Q. wutaishanica* forests in different water availability conditions, our study found significant differences in the strategies of the two communities. Economic and hydraulic traits are strongly coordinated at the community level. Functional diversity based on hydraulic traits are different from economic traits. Deterministic assembly pattern was tested based on economic and hydraulic traits. Consideration of economic traits may not be necessary to study community assembly in an arid ecosystem. Environmental filtering acting on hydraulic was especially stronger than that acting on economic traits in lower water availability. A key set of functional traits which are important for understanding the community assembly process should be considered in future community ecology research. Our findings had profound implications for understanding community assembly within different water availabilities in the natural temperate forests of northern China and for more pragmatic matters, such as informing community restoration based on trait-based principles.

## Data Availability Statement

The original contributions presented in the study are included in the article/Supplementary Material, further inquiries can be directed to the corresponding author/s.

## Author Contributions

JZ and MY conceived and designed the experiments. JZ, YZ, JX, YFC, PL, YC, CL, and JGZ performed the experiments. JZ, YZ, and QY analysed the data. JZ and MY wrote the manuscript. All authors contributed to the article and approved the submitted version.

## Conflict of Interest

The authors declare that the research was conducted in the absence of any commercial or financial relationships that could be construed as a potential conflict of interest.

## Publisher’s Note

All claims expressed in this article are solely those of the authors and do not necessarily represent those of their affiliated organizations, or those of the publisher, the editors and the reviewers. Any product that may be evaluated in this article, or claim that may be made by its manufacturer, is not guaranteed or endorsed by the publisher.

## Supplementary Material

Supplementary Figure 1 Barplot comparing soil water content (SWC) among per plot between LP and QL.

Supplementary Figure 2 Phylogenetic dendrogram of study site community species on LP.

Supplementary Figure 3 Phylogenetic dendrogram of study site community species in QL.
