# Supplementary figures and images for: Strong Environmental Filtering Based on Hydraulic Traits Occurring in the Lower Water Availability of Temperate Forest Communities
Source: Front Plant Sci. 2022 Jan 20;12:698878. doi: 10.3389/fpls.2021.698878 (PMC8811132; doi:10.3389/fpls.2021.698878)

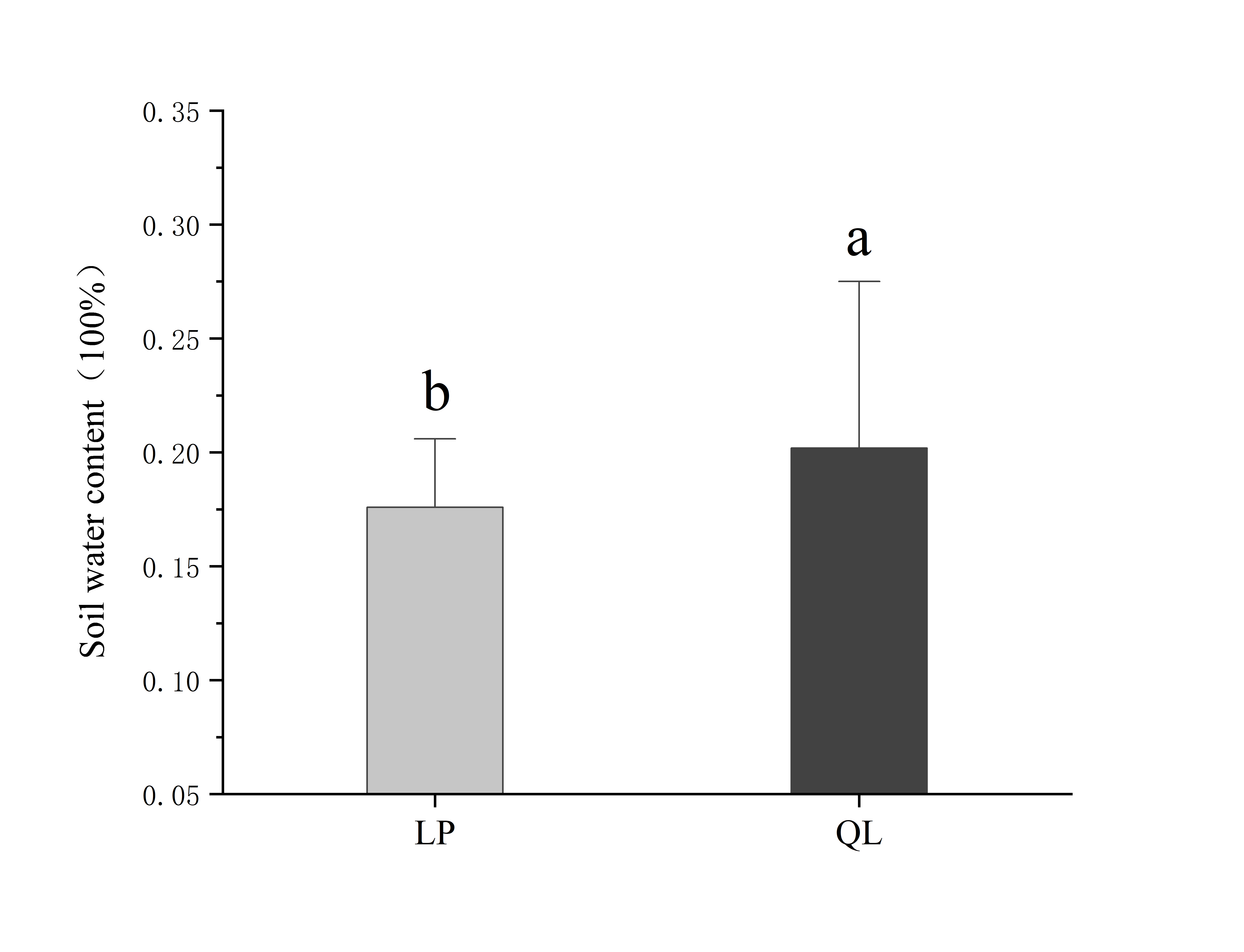

Supplement: Supplementary Figure 1 — Barplot comparing soil water content (SWC) among per plot between LP and QL. [file Image_1.tif]

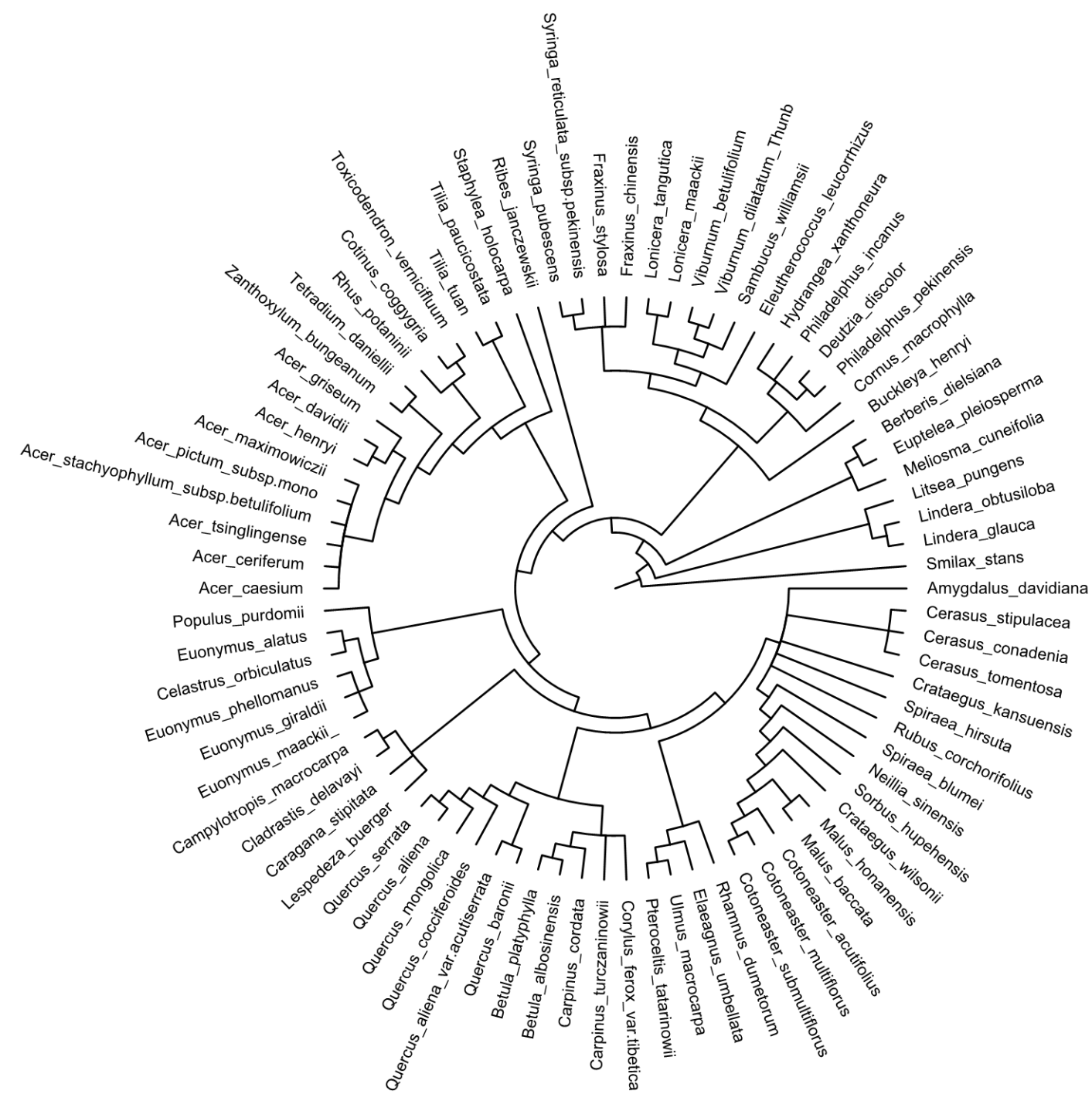

Supplement: Supplementary Figure 3 — Phylogenetic dendrogram of study site community species in QL. [file Image_3.pdf]
