# Supplementary material for: Strong Environmental Filtering Based on Hydraulic Traits Occurring in the Lower Water Availability of Temperate Forest Communities
Source: Front Plant Sci. 2022 Jan 20;12:698878. doi: 10.3389/fpls.2021.698878 (PMC8811132; doi:10.3389/fpls.2021.698878)
Supplement: Supplementary Figure 2 — Phylogenetic dendrogram of study site community species on LP. [file Image_2.pdf]

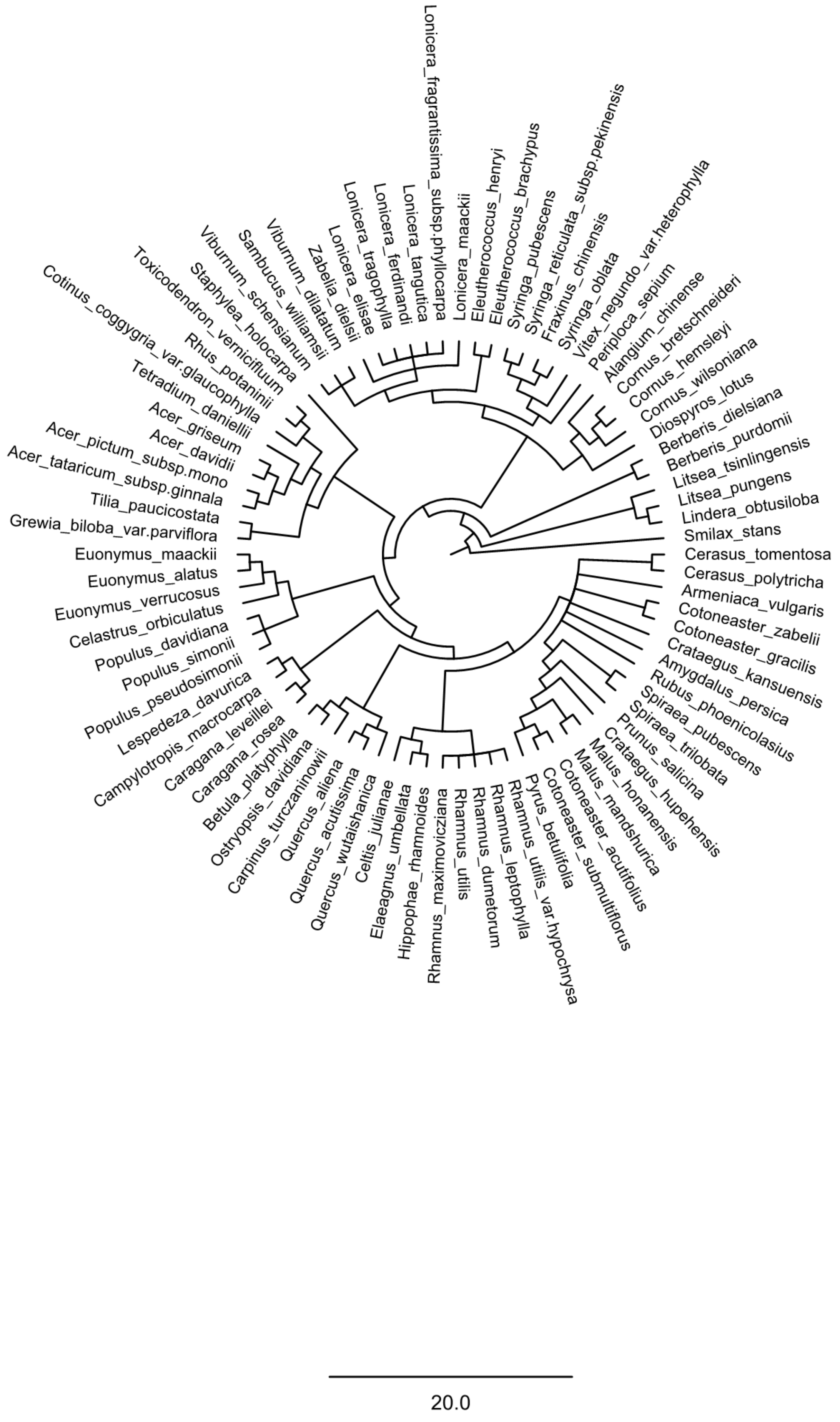
Lonicera_fragrantissima_subsp.phyllocarpa
Lonicera_maackii
Eleutherococcus_henryi
Eleutherococcus_brachypus
Syringa_pubescens
Syringa_reticulata_subsp.pekinensis
Fraxinus_chinensis
Syringa_oblata
Vitex_negundo_var.heterophylla
Periploca_sepium
Alangium_chinense
Cornus_bretschneideri
Cornus_hemsleyi
Cornus_wilsoniana
Diospyros_lotus
Berberis_dielsiana
Berberis_purdomii
Litsea_tsinlingensis
Litsea_pungens
Lindera_obtusiloba
Smilax_stans
Cerasus_tomentosa
Cerasus_polytricha
Armeniaca_vulgaris
Cotoneaster_zabelii
Cotoneaster_gracilis
Crataegus_kansuensis
Amygdalus_persica
Rubus_phoenicolasius
Spiraea_pubescens
Spiraea_trilobata
Prunus_salicina
Crataegus_hupehensis
Malus_honanensis
Malus_mandshurica
Cotoneaster_acutifolius
Cotoneaster_submultiflorus
Pyrus_betulifolia
Rhamnus_utilis_var.hypochrysa
Rhamnus_leptophylla
Rhamnus_dumetorum
Rhamnus_utilis
Rhamnus_maximovicziana
Hippophae_rhamnoides
Elaeagnus_umbellata
Celtis_julianae
Quercus_wutaishanica
Quercus_acutissima
Quercus_aliena
Carpinus_turczaninowii
Ostryopsis_davidiana
Betula_platyphylla
Caragana_rosea
Caragana_leveillei
Campylotropis_macrocarpa
Lespedeza_davurica
Populus_pseudosimonii
Populus_simonii
Populus_davidiana
Celastrus_orbiculatus
Euonymus_verrucosus
Euonymus_alatus
Euonymus_maackii
Grewia_biloba_var.parviflora
Tilia_paucicostata
Acer_tataricum_subsp.ginnala
Acer_pictum_subsp.mono
Acer_davidii
Acer_griseum
Tetradium_daniellii
Cotinus_coggygria_var.glaucophylla
Rhus_potaninii
Toxicodendron_vernicifluum
Staphylea_holocarpa
Viburnum_schensianum
Sambucus_williamsii
Viburnum_dilatatum
Zabelia_dielsii
Lonicera_elisae
Lonicera_tragophylla
Lonicera_ferdinandi
Lonicera_tangutica
20.0
